# Impact of early parenteral nutrition on catabolism

**DOI:** 10.1186/cc12193

**Published:** 2013-03-19

**Authors:** J Gunst, I Vanhorebeek, MP Casaer, G Hermans, PJ Wouters, J Dubois, K Claes, M Schetz, G Van den Berghe

**Affiliations:** 1 KU Leuven, Belgium; 2 Jessa Hospital, Hasselt, Belgium

## Introduction

Prolonged critically ill patients enter a state of hypercatabolism and muscle weakness, which has been associated with increased morbidity and mortality. Early, full feeding of ICU patients has been advocated to counteract catabolism. However, a large, multicenter study found that early parenteral nutrition (PN) had no significant impact on mortality and even increased dependency on intensive care with, among others, a significant prolongation of the duration of renal replacement therapy (RRT) [1]. The impact of the intervention on early markers of catabolism has not been investigated.

## Methods

We studied the impact of early versus late PN on daily markers of catabolism in the ICU in the total study population and in propensity score-matched subgroups of long-stay patients. In addition, we calculated the net incorporation rate of the extra amino acids supplied by early PN.

## Results

Plasma urea, the urea/creatinine ratio and nitrogen excretion increased over time in the ICU. Early PN further increased these markers of catabolism, from the first day of amino acid infusion onward, and only marginally improved the nitrogen balance. Also in the group that received PN only after the first week in the ICU, ureagenesis was increased by infusing amino acids. Over the first 2 weeks, approximately two-thirds of the extra amino acids supplied by early PN were net wasted in urea. The above findings were confirmed in propensity score-matched subgroups of long-stay patients. The higher urea levels with early PN, rather than the kidney function as such, may have driven the observed longer duration of RRT, as supported by multiple regression analysis.

## Conclusion

The extra amino acids supplied by early PN appeared inefficient to reverse the negative nitrogen balance, not because of insufficient amino acid delivery, but rather because of insufficient incorporation with, instead, increased degradation into urea. The substantial catabolism of the extra amino acids, leading to pronounced urea generation, may have prolonged the duration of RRT in the early PN group.
